# News and Coming Events

**Published:** 1908-11-14

**Authors:** 


### News and Coming Events.

The Nurses' Registration Bill passed its third reading in the House of Lords on Tuesday, November 10.

Princess Henry of Battenberg has signified her intention of being present at the matinée to be given at the Playhouse Theatre on November 26 in aid of the funds of the Infants Hospital, Vincent Square, Westminster.

The Duke and Duchess of Connaught have made a donation of £800 towards the equipment of a new hospital for the island of Malta, which will be established at Città Vecchia in place of the military hospital, and will be known as the Connaught Hospital.

Dr. S. R. and Mrs. Collier, the retiring Mayor and Mayoress of Wimbledon, were entertained on November 7 to a complimentary dinner by the Corporation, officials, members of the Education Committee, and other committees associated with the municipality.

At the last general Court of the Governors of St. Bartholomew's Hospital, held on Thursday, November 5, Lord Sandhurst was unanimously elected treasurer of the hospital, in place of Lord Ludlow, resigned. Lord Sandhurst was also formally admitted a Governor of the institution.

The 126th anniversary of the Nottingham Hospital was celebrated on October 28. The customary meeting of governors and subscribers was preceded by a special service in St. Mary's Church, where the Rev. H. Hensley Henson, D.D., Canon of Westminster, preached an eloquent sermon.

During the forthcoming season a grand bazaar and fête will be held in aid of the Children's Hospital in Great Ormond Street. Encouraged by the great success of the Coronation bazaar in 1902, an influential committee, headed by Lady Pembroke, have decided to repeat their experiment next summer.

The Great Northern Central Hospital is at present suffering from a serious diminution of income, amounting to £6,000 for the current year. The annual expenditure averages £17,000, and the total debt to the bankers amounts to over £12,000. Unless this debt can be reduced and funds obtained for maintenance the committee will be faced with the necessity of closing a ward.

The Daimler car which was exhibited at the last Godiva procession at Coventry, and which had been presented to the Coventry and Warwickshire Hospital by the Daimler Co., has been purchased by a committee representative of the counties of Worcester and Warwickshire, and presented to the Bishop of the Diocese, and the proceeds will be applied to the Extension Fund of the Hospital.

In reply to a letter from Sir Alfred Jones, informing him of the departure of Professor Newstead and Dr. Prout for the West Indies to investigate the malaria-carrying insects, Mr. Chamberlain writes expressing his satisfaction that the Liverpool School of Tropical Medicine is continuing its activity, and wishes all success to its representatives, Professor Newstead and Dr. Prout, in their work.

The Khedive of Egypt has been pleased to confer upon Professor William R. Smith, D.L., J.P., M.D., Principal of the Royal Institute of Public Health, the Star of Commander of the Imperial Order of the Medjidieh, and upon Mr. James Cantlie, M.A., M.D., honorary secretary of the Royal Institute of Public Health, the fourth class of the Imperial Order of the Osmanieh, in recognition of services rendered to his Highness.

Sir A. Conan Doyle, M.D., will preside at the eighteenth annual smoking concert arranged by the students of the Middlesex Hospital, at the Queen's Hall, on the 24th inst. The proceeds will be given to the Middlesex Hospital Cancer Wards. Many leading artists of the theatrical and music-hall stages have consented to appear. Tickets may be obtained from the honorary secretaries at the hospital or at the Queen's Hall.

After considerable discussion, the City Board of Guardians decided on November 10 to reconsider their decision of a month ago, and to continue their subscription of twenty guineas to the London Hospital. Opposition was offered on the ground that there was a similar institution almost next door to the union offices which contributed to the City poor rate and received nothing, and that a large part of the income of the London Hospital was derived from the City Companies and City merchants. The rescinding motion was carried by eighteen votes to nine, a two-thirds majority being necessary.

At the Liverpool Police Court a man named Dovsky was recently sentenced to a month's imprisonment and heavily fined for having corruptly offered money to Dr. David John Young, surgeon on the White Star liner *Cymric*, as an inducement to him to pass certain intending emigrants. It was stated that he offered Dr. Young sums ranging from £2 to £25 to pass at this side of the Atlantic a number of intending emigrants, who, upon examination by the doctor, were found to be suffering from trachoma. Police testimony was offered to the effect that the prisoner had been "trafficking for years past in rejected emigrants." Dovsky was recommended for deportation under the Aliens Act.

Sir Henry Alfred Pitman, M.D., F.R.C.P., died on November 6 at Enfield at the age of 100. Born in 1808, he was educated at Trinity College, Cambridge, and at St. George's Hospital, where he was assistant physician from 1857 to 1866. He was Registrar of the Royal College of Physicians from 1858 till 1889, in which year he became Emeritus Registrar; and he was knighted in 1883. From 1876 to 1886 Sir Henry represented the Royal College of Physicians of London on the General Medical Council. When he reached his 100th birthday, in July of this year, he received countless congratulatory messages, together with a handsome bowl, while the King telegraphed his cordial congratulations.

The Earl of Dartmouth has issued an appeal on behalf of the Miller General Hospital for South-East London. It points out the deficiency in hospital accommodation in South-East London as compared with other districts. The hospital serves an area in which is included the second poorest group of 90,000 persons in the whole of the Metropolis. It has only 25 beds available for its nearly 20,000 patients per year, and in the four surrounding boroughs there is a population of close upon half a million. It is believed that 200 beds could be filled with deserving hospital patients. The present annual expenditure is about £4,500, but from the figures of hospitals in other districts it would appear that £15,000 to £20,000 per annum is required to support a hospital at all adequate to the needs of the district. The extension, if sufficiently supported, would enable the Board to provide the accommodation that is so urgently needed for children.

